# The Biosafety Research Road Map: The Search for Evidence to Support Practices in the Laboratory—Crimean Congo Haemorrhagic Fever Virus and Lassa Virus

**DOI:** 10.1089/apb.2022.0044

**Published:** 2023-12-05

**Authors:** Stuart D. Blacksell, Sandhya Dhawan, Marina Kusumoto, Kim Khanh Le, Kathrin Summermatter, Joseph O'Keefe, Joseph Kozlovac, Salama Suhail Almuhairi, Indrawati Sendow, Christina M. Scheel, Anthony Ahumibe, Zibusiso M. Masuku, Allan M. Bennett, Kazunobu Kojima, David R. Harper, Keith Hamilton

**Affiliations:** ^1^ Mahidol-Oxford Tropical Research Medicine Unit, Faculty of Tropical Medicine, Mahidol University, Bangkok, Thailand.; ^2^ Centre for Tropical Medicine and Global Health, Nuffield Department of Medicine, Nuffield Department of Medicine Research Building, University of Oxford, Oxford, United Kingdom.; ^3^ Institute for Infectious Diseases, University of Bern, Bern, Switzerland.; ^4^ Ministry for Primary Industries, Wellington, New Zealand.; ^5^ U.S. Department of Agriculture, Agricultural Research Service, Beltsville, Maryland, USA.; ^6^ Abu Dhabi Agriculture and Food Safety Authority, Abu Dhabi, United Arab Emirates.; ^7^ Research Center for Veterinary Science, National Research and Innovation Agency, Indonesia.; ^8^ WHO Collaborating Center for Biosafety and Biosecurity, Office of the Associate Director for Laboratory Science, Center for Global Health, Centers for Disease Control and Prevention, Atlanta, Georgia, USA.; ^9^ Nigeria Centre for Disease Control, Abuja and Prevention, Nigeria.; ^10^ National Institute for Communicable Diseases of the National Health Laboratory Services, Sandringham, South Africa.; ^11^ UK Health Security Agency, Porton Down, United Kingdom.; ^12^ Department of Epidemic and Pandemic Preparedness and Prevention, World Health Organization, Geneva, Switzerland.; ^13^ The Royal Institute of International Affairs, London, United Kingdom.; ^14^ World Organisation for Animal Health (OIE), Paris, France.

**Keywords:** Crimean Congo Haemorrhagic Fever virus, Lassa virus, pathogen characteristics, biosafety evidence, knowledge gap analysis

## Abstract

**Introduction:**

Crimean Congo Hemorrhagic Fever (CCHF) virus and Lassa virus (LASV) are zoonotic agents regarded as high-consequence pathogens due to their high case fatality rates. CCHF virus is a vector-borne disease and is transmitted by tick bites. Lassa virus is spread via aerosolization of dried rat urine, ingesting infected rats, and direct contact with or consuming food and water contaminated with rat excreta.

**Methods:**

The scientific literature for biosafety practices has been reviewed for both these two agents to assess the evidence base and biosafety-related knowledge gaps. The review focused on five main areas, including the route of inoculation/modes of transmission, infectious dose, laboratory-acquired infections, containment releases, and disinfection and decontamination strategies.

**Results:**

There is a lack of data on the safe collection and handling procedures for tick specimens and the infectious dose from an infective tick bite for CCHF investigations. In addition, there are gaps in knowledge about gastrointestinal and contact infectious doses for Lassa virus, sample handling and transport procedures outside of infectious disease areas, and the contribution of asymptomatic carriers in viral circulation.

**Conclusion:**

Due to the additional laboratory hazards posed by these two agents, the authors recommend developing protocols that work effectively and safely in highly specialized laboratories in non-endemic regions and a laboratory with limited resources in endemic areas.

## Introduction

The World Organization for Animal Health, World Health Organization (WHO), and Chatham House are collaborating to improve the sustainable implementation of laboratory biological risk management, particularly in low-resource settings. The Biosafety Research Roadmap (BRM) project aims at supporting the application of laboratory biological risk management and improving laboratory sustainability by providing an evidence base for biosafety measures (including engineering controls) and evidence-based biosafety options for low-resource settings.

This will inform strategic decisions on global health security and investments in laboratory systems. This work involves assessing the current evidence base required for implementing laboratory biological risk management, aiming at providing better access to evidence, identifying research and capability gaps that need to be addressed, and providing recommendations on how an evidence-based biorisk management approach can support biosafety and biosecurity in low-resource settings.

## Methods

A technical working group (TWG) was formed to develop a BRM to support the application of laboratory biological risk management and improve laboratory sustainability by providing an evidence base for biosafety measures. The TWG conducted a gap analysis for a selected list of priority pathogens on procedures related to diagnostic testing and associated research for those pathogens, including but not limited to sample processing, testing, animal models, tissue processing, necropsy, culture, storage, waste disposal, and decontamination.

The TWG screened databases, websites, publications, reviews, articles, and reference libraries for relevant data to achieve this. The main research domains used to perform the literature searches were the ABSA database, Belgian Biosafety Server, US centres for disease, control and prevention reports, WHO reports, PubMed, and internet searches for terms related to biosafety matters, including, for example, inactivation, decontamination, laboratory-acquired infections (LAIs), laboratory releases, and modes of transmission.

The summary of evidence and potential gaps in biosafety was divided into five main sections: route of inoculation/modes of transmission, infectious dose, LAIs, containment releases, and disinfection and decontamination strategies. Blacksell et al. provide a detailed description of the materials and methods and an introduction to why the gap analysis was conducted.^1^

This review details the general characteristics of Crimean Congo Hemorrhagic Fever virus (CCHFV) and Lassa virus, the current biosafety evidence, and available information regarding LAIs and laboratory releases.

## Crimean Congo Hemorrhagic Fever Virus

### General Characteristics

CCHFV is a negative-sense, single-stranded enveloped RNA virus belonging to the family *Bunyaviridae*.^2–4^ Several genera of *Ixodid* ticks serve as vectors and reservoirs for CCHFV, with ticks in the genus *Hyalomma* being of primary importance. Other tick genera involved in CCHFV ecology include *Rhipicephalus*, *Ornithodoros*, *Boophilus*, *Dermacentor*, and *Ixodes*.^5^ Implicated mammalian reservoirs include wild hares, hedgehogs, rodents, and agriculturally important species, including cattle, sheep, swine, and goats.

Although many birds are resistant to infection, CCHFV has been reported to cause a disease outbreak in ostriches and migratory birds. Crimean Congo Hemorrhagic Fever (CCHF) has an overall case fatality rate between 10% and 40%,^6^ and in documented episodes of hospitalized patients, the fatality rate can be as high as 50%.^7^ The onset of symptoms is sudden and commonly includes headache, high fever, back and joint pain, stomach pain, and nausea.

Pinpoint bleeding results in a reddening of the eyes and the formation of red spots in the palate and throat. As the hemorrhagic disease progresses, severe bruising and nosebleeds, uncontrolled bleeding at injection sites, and multiorgan failure and shock can lead to death.

CCHFV is categorized as Risk Group 4 by the Public Health Agency of Canada and the European Commission^8,9^ and as a select agent by the United States Federal Select Agent Program.^10^

#### Treatment and prophylaxis

Treatment for CCHF is primarily supportive, including maintaining fluid and electrolyte balance, oxygenation, hemodynamic support, treatment of secondary infections, and oral ribavirin. Oral ribavirin is also used as post-exposure prophylaxis for those who have come in contact with highly viremic patients.^11^ Prevention of exposure in endemic environments is via control of tick populations using insecticides,^4^ adhering to proper infection control precautions to prevent occupational exposure in healthcare settings, and wearing protective clothing and tick repellent when working with susceptible animal species.^12^

#### Diagnosis

Laboratory tests used to diagnose CCHF include reverse transcriptase-polymerase chain reaction (RT-PCR), immunofluorescence assay, antibody (immunoglobulin G, immunoglobulin M) and antigen-capture enzyme-linked immunosorbent assay (ELISA), and virus isolation.^13^ RT-PCR is the primary diagnosis for patients suspected of having CCHF, as these assays provide the highest detection sensitivity to active infection at the earliest time point.^13^ Virus isolation is rarely used as a diagnostic tool because of the requirement for maximum biosafety level (BSL4) biocontainment practices and principles^13^ and time to diagnosis.

##### Biocontainment considerations pertinent to diagnostics

In areas where CCHF is non-endemic, work with CCHFV is usually performed under maximum containment conditions. Conversely, in countries where CCHF is endemic, very few maximum containment laboratories exist due to the high construction, maintenance, and operation cost. As rapid diagnosis is of great importance, this work is typically carried out in Core (or BSL-2) laboratories using Enhanced Control Measures to manage risks associated with aerosol generation and waste management.

Enhanced Control Measures include using a biological safety cabinet (BSC) to manipulate samples suspected of containing virus, adherence to best practices to reduce the inadvertent generation of aerosols, and decontaminating wastes before removal from the BSC. Decontamination methods should be validated before waste disposal. In some countries, donning gloves is not a requirement in diagnostic labs. As CCHFV can be transmitted by contact to abrasions, cuts, and other wounds to the skin, the use of gloves is recommended when handling samples and performing diagnostic assays until the virus is known to be inactivated (i.e., by chemicals used in the procedure).

### Biosafety Evidence

#### Modes of transmission

CCHF is commonly transmitted via infected tick bites, exposure to infectious blood or secretions, needlestick injuries, contact with contaminated fomites, and inadvertent contact with skin lesions.^3,11^ There is evidence of probable aerosol transmission associated with nosocomial infection of eight healthcare workers (HCWs) in Russia.^14^ It is endemic to most southeast Europe, Asia, the Middle East, and Africa. It is a significant occupational hazard for farmers, veterinarians, medical personnel, and abattoir workers living in endemic areas.^15^

#### Infectious dose

Natural CCHFV infection occurs following an infected tick bite. The infectious dose has not been determined in natural or laboratory exposures. However, a combined subcutaneous and intravenous dose of 5 log10 tissue culture infectious doses (TCID)_50_ was needed to cause disease (sometimes fatal) in nonhuman primates.^16^

#### Laboratory-acquired infections

A retrospective analysis of 158 CCHF nosocomial infections between 1953 and 2016 indicated that 92.4% were symptomatic, with 8% (10) of those considered LAIs.^17^ In 2003, Turkey experienced an epidemic of CCHF, with 9787 laboratory and clinically confirmed human infections.^18^ A retrospective study demonstrated that 51 healthcare-related exposures occurred mainly by needlestick (62.7%), mucous membrane splash with blood/body fluid (23.5%), and 13% unidentified routes of exposure. The infections resulted in 4 deaths, and the authors reference the need for sharps safety and personal protective equipment (PPE) training to improve exposure prevention and control.^19^

It is estimated that 90,000–100,000 patient specimens were processed under BSL-2 conditions.^19^ Despite the large volume of specimens handled, only two cases in laboratorians were reported; although formally reported as unknown routes of exposure, one may have occurred while drawing blood from a CCHF patient and the other while handling a blood sample without wearing gloves.^19^ In South Africa, 214 cases of CCHF have been confirmed since the disease was recognized, and 811 acute-phase blood samples have been handled in the reference laboratory under BSL-2 or BSL-3 conditions without any reports of LAI.^18^

The HCWs have a far higher risk of acquiring CCHF and account for ≥86%^17^ of all accidental (non-wild-type) symptomatic infections. Most cases are caused by either needlestick or lack of appropriate PPE and direct contact with infected body fluids. One prospective study indicated that only about half of HCWs exposed to body fluids containing CCHF acquired the disease following adequate remedial measures taken after exposure (flushing, rinsing, etc.).^18^

This study likely captured exposures that would not have been reported elsewhere, which may account for the disagreement with Tsergouli et al.^17^ Lastly, secondary transmission in non-HCWs does occur in hospitals and accounts for 13% of nosocomial infections.^17^ Most cases are among family members who have visited acutely ill patients, sat within close proximity, and/or had physical contact.

#### Decontamination and inactivation

##### Chemical

Although Bartolini et al.^28^ state, “Like all lipid-enveloped viruses, CCHFV can be readily inactivated by common fixatives such as 2% glutaraldehyde,^20^ formalin and paraformaldehyde; 1% sodium hypochlorite^20,21^” or low pH (less than 6),^22^ they do not provide contact times used during inactivation. As the primary references also did not provide contact times, the guidance for inactivating CCHFV is incomplete. Although 70% ethanol is typically recommended as a decontaminant for susceptible viruses, in one study, CCHFV was inactivated in 40% ethanol within 2 min.^23^

A critical aspect of laboratory biosafety and operation is the proper and reliable inactivation of specimens before they can be removed from the high-level biocontainment environment for further diagnostic testing.

Further peer-reviewed published evidence for the effectiveness of inactivation procedures is needed, especially for their impact on clinical laboratory tests. Although there is no direct evidence of its effects on CCHFV, treatment with Triton X-100 can decrease the biohazard risk of performing laboratory tests on samples from patients infected with other hemorrhagic fever viruses (i.e., Ebola) without affecting the results of biochemical tests.^24–26^ 4′-Aminomethyl-trioxsalen at 10 μg/mL is also effective in inactivating CCHFV.^27^

##### Thermal and autoclaving

Physical inactivation is also effective, such as high temperature (56°C for 30 min or 60°C for 15 min).^18,20,22,28^ Although autoclave cycles employing 121°C for 30 min minimum sterilization time are typically used in the laboratory for waste containing other enveloped viruses, cycles require validation for different load types and sizes.

##### Radiation

Examination of the inactivation of CCHF in biological samples revealed that the infectivity of plasma-reduced platelets was reduced by a factor of ≥2.2 log_10_ to the limit of detection (LOD) when irradiated with ultraviolet C (UVC) at a wavelength of 254 nm to a dose of 0.1 J/cm^2^, whereas CCHV in plasma treated with methylene blue and light emitting diode-based light energy (30 J/cm^2^) was reduced by a factor of ≥3.2 log_10_ to below the LOD.^29,30^ Irradiation with UV-A at 4000 μW/cm^2^ at 20 min exposure^27^ and (1200–3000 μW/cm^2^)^28^ has also proven effective.

Evidence regarding the route of inoculation/modes of transmission, infectious dose, LAIs, and disinfection and decontamination strategies is provided in Table 1.

**Table 1.** Detailed pathogen biosafety evidence for Crimean-Congo hemorrhagic fever virus

Overview of the evidence and potential gaps in biosafety				
Method	Details	Evidence (direct quote where available)	Reference	Evidence gap? (yes/no)
Route of inoculation	Cutaneous/percutaneous	“Community-acquired CCHF happens through transmission of the virus by direct contact with blood or other infected tissues of livestock or from an infected tick bite…… CCHFV has repeatedly caused nosocomial outbreaks with high mortality, and percutaneous exposure presents the highest risk of transmission”	^11^	No
		“Humans can be infected incidentally by the bite of an infected arthropod.” “The primary transmission route was percutaneous contact (34.3%). Cutaneous contact accounted for 22.2% of cases”	^17^	
		“Tygerberg Hospital in 1985, of 459 listed contacts, 4 out of 46 blood contacts (8.7%) and 3 out of 9 needle contacts (33%) developed the disease. It is not apparent that blood contacts were with intact skin or mucosa.^6^ In a report from Pakistan in 1998, two of four people exposed percutaneously and one of five with cutaneous exposure contracted CCHF”	^61^	
		“Harxhi and others, in an outbreak in Albania, concluded that the CCHF agent can be transmitted through apparently intact skin exposed to infected blood but, in the absence of skin defects or percutaneous injury with a contaminated device, exposure of mucous membranes through droplets or contaminated hands could have played a more important role”	^61^	
		“Human beings become infected through tick bites, by crushing infected ticks, after contact with a patient with CCHF during the acute phase of infection, or by contact with blood or tissues from viraemic livestock”	^15^	
		“The main routes of exposure were needlestick injury (NSI) in 32/51 (62.7%), defined blood/bodily fluid exposure to mucous membranes (splash) in 12/51 (23.5%) and unidentified in 7/51 (13.7%)”	^19^	
	Inhalation	“Humans can be infected incidentally by the bite of an infected arthropod or via aerosol generated from infected rodents' excreta”	^11^	No
		“The primary transmission route was percutaneous contact (34.3%). Cutaneous contact accounted for 22.2% of cases, followed by exposure to aerosols (proximity) (18.2%)”	^17^	
		LAI Case in Kazahkstan (1973)—“It was concluded that mixing volatile freon with the brain suspension may have caused formation of aerosols which were inhaled”	^18^	
		LAI Case in Rostov-na-Donu, Russia (1970) “A flask with highly active virus-containing material was broken in a centrifuge rotor, which probably led to infection through aerosol”	^62^	
Infectious dose	Unknown	There is a lack of a clearly defined animal model, agreed upon CCHFV strain for use, dose level (ID or LD), dose measure (TCID or PFU), or mode of inoculation	^32^	Yes
LAIs	1 case in Rostov-na-Donu, Russia (1968)	“In summer of 1968 in Rostov-na-Donu while visiting the Institute of Epidemiology, Microbiology and Hygiene, a laboratory assistant from Institute of Poliomyelitis and Viral Encephalitis was infected while processing a blood specimen from a patient with CCHF (centrifuging and preparation of plasma for infecting mice)”	^62^	No
	1 case in Rostov-na-Donu, Russia (1970), resulting in death	“On February 20, 1970, at Rostov-na-Donu Institute of Epidemiology, Microbiology, and Hygiene one more laboratory assistant fell ill. She and 3–4 staff members worked with live CCHF virus 10 days before the onset of the disease. A flask with highly active virus-containing material was broken in a centrifuge rotor, which probably led to infection through aerosol, although other workers present in the same room did not fall ill”	^62^	
	8 cases in Uganda (1960s)	“Eight laboratory infections, one fatal, were recorded in Uganda during early investigations of ‘Congo’ virus in the 1960s, where known exposure of patients to infection occurred during the handling or processing of infected mice”	^18^	
	1 case in Egypt (1981)	“In 1981, a virologist died in Cairo, Egypt, after mouth-pipetting a culture of a CCHFV isolate he had brought from Iraq”	^18^	
	3 cases in Senegal (1998, 1993)	“At the Institute Pasteur de Dakar, two accidents were linked to handling suckling mice inoculated with a diagnostic sample and a tick pool suspension: in 1998, a technician suffered a needlestick accident, and in 1993, a staff member in breach of regulations handled cages with infected mice on an open bench without wearing any mask….Also in 1993, another technician was exposed to aerosols while preparing sucrose acetone antigen from infected suckling mouse brain since not all equipment was held in a laminar flow cabinet or in a BSL-3 laboratory”	^18^	
	1 case in South Africa (1986)	“In South Africa, a clinical pathology laboratory technologist in a hospital in Kimberly was found to be seropositive for CCHF in 1986….The technologist routinely wore a laboratory coat and disposable gloves and performed all manipulations with blood and serum in class II cabinets”	^18^	
	1 case in South Africa (2006)	“A fatal case of CCHF occurred in 2006 in a technologist in a clinical pathology laboratory in Vereeniging, South Africa, who putatively only handled blood samples from a deceased CCHF patient in order to store them in a freezer. He had signed a procedure protocol which instructed him to wear a laboratory coat and gloves, but nobody observed him storing the samples. The technologist reportedly had not tested the samples, and it was never determined whether he had worn gloves or how he was exposed to infection, but virus isolates from the source patient and the technologist had identical nucleotide sequences”	^18^	
	1 case in Kazakhstan (1973)	“In 1973, at the Institute for Epidemiology, Microbiology and Infectious Disease in Alma Ata (USSR, now Kazakhstan), a scientist preparing CCHFV antigen from suckling mouse brain using freon extraction, fell severely ill and seroconverted but recovered. It was concluded that mixing volatile freon with the brain suspension may have caused formation of aerosols which were inhaled”	^18^	
	Cases in Turkey	“The two infections that occurred in laboratory staff were included in the unidentified exposure group, although one may have occurred while taking blood from a CCHF patient and the second while handling a blood sample in the laboratory without wearing gloves”	^19^	
	1 case in Bulgaria (1999)	“In 1999, a technician inflicted an abrasion on her hand with a needle during a CCHFV baby mouse brain passage procedure in the National Center of Infectious and Parasitic Diseases laboratory in Sofia, Bulgaria. However, she was vaccinated with the Bulgarian CCHFV vaccine and presented with benign febrile illness only”	^18^	
Chemical inactivation	40% ethanol for 2 min	“In order to test virus inactivation in ethanol, HTNV, SFSV, and CCHFV were added to different concentrations of ethanol in dilution medium to a final volume of 1 ml. The solutions were vortexed and incubated for 2 min at 20°C before being titrated as described above……HTNV, SFSV, and CCHFV were undetectable after 2 min in 40% ethanol”	^23^	Yes
	0.5% sodium hypochlorite 2% glutaraldehyde 0.5–3% phenolic disinfectants	“Lipid-containing viruses, including the enveloped viruses, are among the most readily inactivated of all viral agents. Suitable disinfectant solutions include 0.5% sodium hypochlorite (10% aqueous solution of household bleach), as well as fresh, correctly prepared solutions of glutaraldehyde (2% or as recommended by the manufacturer) and phenolic disinfectants (0.5%–3%). Soaps and detergents can also inactivate these viruses and should be used liberally”	^63^	
	0.5% sodium hypochlorite 2% glutaraldehyde 0.5–3% phenolic disinfectants	“The Center for Disease Control (CDC), advises ‘Suitable disinfectant solutions include 0.5% sodium hypochlorite (10% aqueous solution of household bleach), as well as fresh, correctly prepared solutions of glutaraldehyde (2% or as recommended by the manufacturer) and phenolic disinfectants (0.5%–3%).’ The use of sodium hypochlorite solutions is widely advised in further CDC guidance on managing suspected viral hemorrhagic fever and by the WHO in their guidelines on laboratory diagnosis of EVD”	^21^	
	2% glutaraldehyde, formalin and paraformaldehyde 1% sodium hypochlorite Hydrogen peroxide and peracetic acid.	“Inactivation: Like all lipid-enveloped viruses, CCHFV can be readily inactivated by common fixatives such as 2% glutaraldehyde, formalin and paraformaldehyde; chlorine-based disinfectants, such as 1% sodium hypochlorite; and other disinfectants, such as hydrogen peroxide and peracetic acid”	^28^	
	10 μg/mL AMT	AMT at 10 μg/mL is effective at inactivating CCHFV	^27^	
	TRIzol, formalin, glutaraldehyde, paraformaldehyde, QIAGEN buffer AVL, QIAGEN buffer RLT buffers with ethanol	Ebola virus was effectively inactivated following 10 min contact time in a TRIzol solution (3 parts TRIzol/1 part infected cells in media); overnight exposure to either 7.5% formalin, 2% glutaraldehyde, or 2% paraformaldehyde at 4°C; and 10 min exposure to QIAGEN buffer AVL or QIAGEN buffer RLT buffer followed by 20 min exposure to 100% or 70% ethanol, respectively	^64,65^	
	**General comment**	**Information on chemical inactivation is incomplete, as the references do not provide contact time and in some cases while they describe inactivation of enveloped viruses, it is not specific to CCHFV**		
Thermal inactivation	60°C temperature for 15 min	“1. Virus characterization. In 1967, Chumakov and his colleagues at the Institute of Poliomyelitis and Viral Encephalitides, Moscow, first used new-born white mice (NWM) and rats (NWR) for CCHF virus isolation…. it passed through 220-nm Millipore filter pores, resisted prolonged freezing on dry ice, and lyophilized well, but became inactivated when exposed to 60°C temperature for 15 min”	^22^	No
	56°C for 30 min or 60°C for 15 min	“Physical inactivation is also effective, like high temperature (56°C for 30 min or 60°C for 15 min)”	^28^	
	56°C for only 15 min	“In experiments performed recently in a South African laboratory to clearly analyse the conditions needed to inactivate CCHFV, CCHFV (strain SPU4/81) culture fluid with a titre of 1 × 10^7^.^6^ TCID_50_/ml was incubated at 56°C and 60°C for 15, 30, 45 and 60 minutes and then inoculated into Vero E6 cell cultures. In all instances, virus growth was not detected. To show that the results were not due to the detection limit of the TCID assay at 1 × 10^1^.^5^ TCID_50_/ml, the inactivated suspensions were also inoculated intracerebrally into suckling mice (NIH strain) and all mice survived, even those inoculated with virus inactivated at 56°C for only 15 minutes”	^18^	
	Autoclaving	Autoclave parameters must be developed and validated based on the type of cycle, load and load size. After a thorough risk assessment and validation, the WHO recommends that the following cycle will usually provide sterilization of correctly loaded autoclaves: 3 min holding time at 134°C; 10 min holding time at 126°C; 15 min holding time at 121°C; and 25 min holding time at 115°C	^66^	Yes
UV light inactivation	UV light (1200–3000 μW/cm^2^)	“Ultraviolet (1,200 to 3,000 μW/cm^2^) or low pH (less than 6) …. It is opinion of the experts that further evaluation of inactivation procedures are needed”	^28^	No
	UVC dose 0.1 J/cm^2^	“The results of the infectivity assays demonstrated that UVC irradiation and MB/light dose-dependently inactivated SARS-CoV, CCHFV. In PCs, at half of the full UVC dose (0.1 J/cm^2^) SARS-CoV and CCHFV infectivity levels were below the LOD, while at three-fourth of the full UVC dose (015 J/cm^2^) also NiV infectivity levels were below the LOD (Table 1). In plasma, already at one-fourth of the full light dose (30 J/cm^2^) SARS-CoV, CCHFV and NiV were inactivated to levels below the LOD”	^29,30^	

AMT, 4′-Aminomethyl-trioxsalen; CCHFV, Crimean Congo Haemorrhagic Fever virus; ID; LAIs, laboratory-acquired infections; LD; PFU, plaque forming units; TCID, tissue culture infectious doses; UV, ultraviolet; WHO, World Health Organization; NiV, Nipah virus; LOD, limit of detection.

### Knowledge Gaps

#### Safe handling of CCHF-infected ticks

There are a few publications on procedures for the safe handling of CCHF-infected ticks, except in the context of research in a high-containment setting.^31^

#### Surveillance measures for CCHF

Surveillance measures for CCHF would naturally include field studies with tick collection, but a few publications delineate safe collection and handling protocols. At a minimum, insect repellents with efficacy against ticks and protective clothing should be worn to prevent tick bites during field studies.

#### Infectious dose

The infectious dose of CCHFV causing infection in humans via tick bites or other routes of infection has not been determined.

#### Animal models

Animal models are essential in identifying infectious dose rates via various transmission methods, pathogenicity mechanisms, antiviral treatments' effectiveness, and vaccine development. Currently, there is a lack of a clearly defined animal model, agreed on CCHFV strain for use, dose level (infections dose or lethal dose), dose measure (TCID or plaque forming units [PFU]), or mode of inoculation^32^ Each of these factors impacts the disease outcome, hence the research results, and subsequently their application to research questions.

### Conclusions

Laboratory-acquired CCHFV infections are relatively rare, despite the lack of high and maximum containment laboratories in endemic countries where the pathogen is handled at a higher volume and with greater frequency. This could partly be due to under-reporting exposure and infection and the lack of a global reporting policy and program.

Although most workplace infections occur in the healthcare setting by individuals working closely with patients, patient samples, and other potentially contaminated materials, LAI have also been reported, as have infections in animal husbandry, veterinary medicine, and abattoir settings. In healthcare settings, using engineered safe sharps, providing sharps safety training, and using barrier PPE would reduce exposure to CCHFV.

In professions where animals are handled or during surveillance studies to collect ticks, insect repellents and protective clothing to prevent tick bites could help reduce exposure and infections. This, coupled with eye/face protection (abattoir workers) and care when using sharps, could further reduce potential CCHFV transmission.

We recommend biosafety best practices using a risk-based approach that allows endemic countries to leverage existing resources to diagnose CCHF infections. Optimally, this would be done by RT-PCR. Diagnostic procedures involving samples that are not readily inactivated or are conducted using equipment that may generate an aerosol should be performed in a containment laboratory with inward directional airflow and heightened control measures (BSL-3 practices, primary containment, and PPE) wherever possible. In resource-constrained environments and where access to high-containment laboratories are lacking, research would ideally be conducted with inactivated CCHFV.

## Lassa Virus

### General Characteristics

Lassa fever (LF) is a viral hemorrhagic fever endemic in West Africa. The causative agent, the Lassa virus (LASV), is a member of the *Arenaviridae* family, a group of viruses generally associated with rodent-transmitted diseases in humans.^33^ LASV is a single-stranded double-segmented negative-sense RNA virus^34^ classified as a Risk Group 4 pathogen, and in the United States deemed a Select Agent.^10^ The host range of the virus is multimammate rats (*Mastomys natalensis*) and humans, the former being reservoirs of the disease.^34^

It is most commonly transmitted via aerosolization of dried rat urine, ingestion of the rat itself, direct contact with, or food and water contaminated with rat excretions.^35,36^ Human-to-human transmission is less common in the general population but is often observed in healthcare settings due to a poor index of suspicion, low infectious dose, needlesticks, and lack of, or poor adherence to, PPE use.^37–40^ Up to 80% of LASV infections are mild, with influenza-like symptoms of low fever, general malaise, and headache.

Twenty percent of infected people may progress to serious symptoms, including mucosal bleeding, respiratory distress, vomiting, facial edema, pain in the chest, back, and abdomen. Later stages include neurologic symptoms, shock, multi-organ failure, and death.^41^

#### Treatment and prophylaxis

Currently, no vaccines for the LASV are licensed for use in humans. Although some have shown promise, no vaccine candidate has shown sufficient efficacy in animal models to have entered phase I human studies. Management involves early recognition of infection by diagnostic testing. Patients should be isolated to prevent further transmission, and HCWs should wear PPE when providing patient care as part of effective infection control.

The WHO recommends that HCWs should wear face protection (a face shield or a medical mask and goggles), a clean, non-sterile long-sleeved gown, and gloves (sterile gloves for some procedures) when working within 3 feet of patients or when handling blood, body fluids, or other potentially contaminated materials (i.e., bedding, patient's clothing).^42^ Patient's contacts within 21 days of the start of symptoms should be traced for monitoring and/or treatment and prophylaxis.

Treatment involves early initiation of the antiviral drug ribavirin and supportive care in the hospital. Supportive care includes maintaining appropriate fluid and electrolyte balance, oxygenation and blood pressure, and treating secondary infections. Ribavirin is occasionally used as post-exposure prophylaxis for high-risk exposure to LASV though its effectiveness has not been definitively determined.^34,43^

#### Diagnosis

The initial investigation in all suspected patients should be RT-PCR for LASV.^44,45^ The highest viremia occurs 4 to 9 days after the onset of symptoms. Serological testing using IgM ELISA should also be carried out. IgM ELISA has 88% sensitivity and 90% specificity for acute infection.^45,46^ If the initial laboratory investigations are negative and clinical suspicion remains high, repeating the laboratory investigations after 24 h could be considered.^45,46^ Due to the low infectious dose via the aerosol route and the potential for severe disease, in vitro isolation of LASV is not recommended in diagnostic laboratory settings.

### Biosafety Evidence

#### Modes of transmission

The natural reservoir of the LASV is the multimammate rat (*M. natalensis*), a rodent found commonly in rural areas of tropical Africa that often colonizes in or around human homes where food is stored.^33^ Transmission of the virus from rodent to human occurs via multiple routes. These include human ingestion of excreta from an infected rodent,^33^ butchering and eating infected rodents (subsistence farmers routinely encounter the rodent when burning their fields ahead of planting and consider it a cheap source of protein), viral exposure to open cuts or sores,^33,35,36,47,48^ or less likely, the inhalation of air contaminated with infected rodent excretions (e.g., aerosolization of rat excreta during sweeping).^33,35,47,49,50^ Infected rodents are asymptomatic and shed the virus in urine throughout their life.^33^

#### Infectious dose

Cynomolgus monkeys exposed to inhaled doses of 465 PFU were infected and died, whereas the ID_50_ for guinea pigs was 15 PFU.^48^ However, the infectious dose varies between LASV strains, animal models, and route of exposure. In comparing animal models, pathology in rhesus and cynomolgus macaques is most similar in humans. The LD_60_ has been demonstrated in rhesus monkeys receiving 10 ^6.1^ PFU of LASV (strain Josiah) by injection^51^ and 1000 PFU of LASV Josiah via aerosol in a head-only chamber in a class III BSC.^52^

#### Laboratory-acquired infections

Only one laboratory-acquired case of LF from a research institute has been reported in the literature in the United States.^53^ The HCW are an at-risk population for contracting LASV after exposure to the virus in the blood, tissue, secretions, or excretions of an LASV-infected individual, especially when standard barrier precautions (gloves, long-sleeved gowns, and face shields or masks and goggles) and patient isolation are not applied.

The 2018 LF outbreak in Nigeria resulted in 8% of all cases occurring among HCWs. In a cluster of cases involving 16 HCWs, 5 were asymptomatic, and 11 developed symptoms resulting in 5 deaths. One of the HCWs worked in the diagnostic laboratory. The authors indicate the lack of diagnosis or suspicion that the index case may have had LF. The resulting HCW infections were due to a lack of PPE use and infection control/prevention procedures and possibly inappropriate isolation of the patient.^54^

#### Decontamination and inactivation

##### Chemical

Chemical inactivation is effective, with 15-min exposure to 3% acetic acid^55^ or 0.2% β-propiolactone at 37°C for 30 min or 4°C for 20 h to inactivate LASV.^56^ Enveloped viruses are typically more susceptible to chemical decontaminants than many non-enveloped viruses and bacteria. Although no chemical inactivation data were found specifically for LASV, chemical inactivation of enveloped viruses has been reported by exposure to 70% alcohol for 1 min, 0.05% sodium linear alkylbenzene sulfonate (anionic surfactant) for 10 min, concentrations and exposure time for povidone-iodine varied with viruses from 0.23% for 15 s to 8% for 30 s, and 4% formaldehyde for 3 h.^57^ Sodium hypochlorite has been demonstrated to inactivate enveloped viruses at a concentration of 0.5% (5000 ppm) for 1 min.^58^

##### Gaseous fumigation

Interior spaces can be decontaminated by formaldehyde gas fumigation (formalin 0.5 mL/ft^3^ of space or paraformaldehyde powder 0.3 gm/ft^3^ of space).^59^ Ideally, formaldehyde fumigation should be conducted at an ambient temperature of at least 70°F (21°C) and a relative humidity of 60%. The gas should remain in contact with the contaminated area for at least 4 h.^59^

##### Thermal and autoclaving

The complete inactivation of LASV has been demonstrated following heating for 60 min at 60°C.^55^ In a different study,^56^ the time-required to inactivate 5 logs PFU/mL of LASV in serum was 37 min. The recommended procedure of heating serum is 60°C for 60 min, as some hemorrhagic fever viruses require longer exposure to heat than others. It should not be assumed that patients are not co-infected or infected with a virus that requires reduced heating time for inactivation.^56^

Although autoclave cycles employing 121°C for 30 min minimum sterilization time are typically used in the laboratory for waste containing other enveloped viruses, cycles have to be validated for different load types and sizes containing LASV.

##### Radiation

Short-wave UV light inactivated LASV successfully after 20 min of exposure to a Mineralight Lamp adjusted to deliver 1200–2000 W/cm^2^.^60^ LASV can be inactivated by gamma irradiation (1.2 × 106 rads to 1.27 × 106 rads).^60^

Evidence regarding the route of inoculation/modes of transmission, infectious dose, LAIs, and disinfection and decontamination strategies is provided in Table 2.

**Table 2.** Detailed pathogen biosafety evidence for Lassa fever

Overview of the evidence and potential gaps in biosafety				
Method	Details	Evidence (direct quote where available)	Reference	Evidence gap? (yes/no)
Route of inoculation	Inhalation	“Virus transmission is believed to occur through aerosols, and the infection of laboratory animals with aenosolized virus has added experimental proof to this assumption. Aerosol transmission also seems to have taken place between patients in hospitals”	^50^	No
		“The two nurses who in 1969 contracted the illness from their respective patients are presumed to have become infected, possibly via respiratory secretion”	^49^	
		“Epidemiologic evidence and the occurrence of nosocomial hospital infections, however, suggest that another possible means of virus dissemination is by airborne droplet nuclei”	^48^	
		“Finally, prolonged presence of high virus titers in the lungs and upper respiratory tract tissues, pharyngeal secretions, and urine of infected humans or rodents would provide exemplary seed sources for infectious aerosols”	^48^	
		“…the inhalation of air contaminated with infected rodent excretions (for example, aerosolisation of rat excreta during sweeping)”	^33^	
	Direct contact	“Transmission by direct person to person contact can occur via virus in blood, pharyngeal secretions, and urine of patients”	^48^	No
		“The virus is excreted in urine for three to nine weeks from infection and in semen for three months. The extent of sexual transmission is unknown”	^36^	
		“Person-to-person transmission of Lassa fever can also occur through contaminated medical equipment, such as reused needles or when a person comes into contact with virus in blood, tissue, secretions, or excretions of an infected individual but virus cannot be spread through casual contact (including skin-to-skin contact without exchange of body fluids)”	^35^	
		“Reports suggest that the most important means of nosocomial transmission is close contact with patients' blood and secretions, via minor cuts or abrasions in the skin, needle sticks, or trauma from other medical instruments”	^47^	
		“Direct contact with blood, tissue, secretions, or excretions of an infected individual. This is mainly seen in hospitals where protective equipment is not available or inappropriately used by healthcare staff and transmission has been attributed to the re-use of needles between patient.”	^33^	
	Cutaneous	“…viral exposure to open cuts or sores”	^33^	No
	Gastrointestinal	“Humans are infected by contact with the rats or by eating them (they are considered a delicacy and are eaten by up to 90% of people in some areas)”	^36^	No
		“Because of the high titers of Lassa virus that are excreted in the urine by infected *Mastomys natalensi*s, the ubiquity of the animals, and the peaking of Lassa fever during the harvest season, it has been assumed that contamination of food stocks with rodent feces and urine is the main mode of rodent-to-human transmission”	^50^	
		“Lassa virus is inactivated when heated at 60°C for 1hr therefore oral transmission might occur only if the meat is for any reason roasted on boiled for a short period of time”	^50^	
		“…human ingestion of excreta from an infected rodent; butchering and eating infected rodents (subsistence farmers routinely encounter the rodent when burning their fields ahead of planting and consider it a cheap source of protein)”	^33^	
Infectious dose	Variable	The median infectious dose (ID50) for guinea pigs was 15 PFU	^51^	Yes
LAIs	1 case—June 1969 Research institute in New Haven, Connecticut	“…a laboratory -acquired infection in an investigator who worked with tissue cultures and mice infected with this virus. The route of infection remains unknown, but it may have been either by direct contact with animals or tissue-culture material, or possibly air-borne”	^53^	Yes
Chemical inactivation	3% acetic acid for 15 min	“We determined that a 15-min exposure to 3% acetic acid was sufficient to inactivate Lassa, Ebola, and Marburg viruses”	^55^	Yes
	Alcohol, anionic surfactant, povidone-iodine, formaldehyde, sodium hypochlorite	Data specific for LASV were not found, though data were found for other enveloped viruses: 70% alcohol for 1 min, 0.05% sodium linear alkylbenzene sulfonate (anionic surfactant) for 10 min, concentrations and exposure time for povidone-iodine varied with viruses from 0.23% for 15 s to 8% for 30 s, and 4% formaldehyde for 3 h, sodium hypochlorite at a concentration of 0.5% (5000 ppm) for 1 min	^57,58^	Yes
	0.2% BPL	“To obtain undetectable levels of virus it was found necessary to double the BPL concentration to 0.2% at 37°C for 30 min or at 4°C for 20 h inactivate virus”	^56^	No
Gaseous fumigation.	Formaldehyde gas 70°F (21°C) and a relative humidity of 60% for 4 h	“Ideally, formaldehyde fumigation should be conducted at an ambient temperature of at least 70°F (21°C) and a relative humidity of 60%. The gas should remain in contact with the contaminated area for 4 hours”	^59^	No
	Formaldehyde gas 0.5 mL/ft^3^ Paraformaldehyde 0.3 gm/ft^3^	“Interior spaces … can be decontaminated by fumigation with formaldehyde gas, which may be generated by heating formalin (0.5 m l/ft^3^ of space) or paraformaldehyde powder (0.3 gm/ft^3^ of space)”	^59^	
Thermal inactivation	60°C for 37 min	“… found that complete inactivation of the three viruses tested occurred only at 60°C.” “The times required to inactivate 5 logs PFU/ml of Lassa, Ebola, and Marburg viruses in serum were 37, 22, and 37 min, respectively”	^55^	No
	60°C for 60 min	“… established the procedure of heating at 60°C for 60 min serum that is suspected of being infected with any of these viruses, which allows an added margin of safety”	^48^	
	Autoclaving	Autoclave parameters must be developed and validated based on the type of cycle, load, and load size. This information is not available in the literature		Yes
UV light inactivation	1200–2000 W/cm^2^ for 20 min	“Short-wave UV light inactivated Lassa virus successfully after 20-min of exposure to a Mineralight Lamp (model R52; UV Products, Inc., San Gabriel, Calif.) adjusted to deliver 1,200 to 2,000 W/cm^2^”	^60^	No
	Gamma irradiation	Gamma irradiation (1.2 × 106 rads to 1.27 × 106 rads)	^60^	
		“Lassa virus can be inactivated in ultraviolet, gamma irradiation, heating from 56–100°C and pH range between 5.5 and 8.5”	^67^	

BPL, β-Propiolactone; PFU, plaque forming units.

### Knowledge Gaps

#### Infectious dose

There is no information regarding inhalation, cutaneous and gastrointestinal median infectious dose in animal models that exhibit similar pathology to humans.

#### Laboratory-acquired infection

Uncertainty regarding the number of LAIs occurring in endemic countries may be due to the lack of formalized reporting mechanisms and the inability to differentiate workplace-related exposure from community exposure.

#### Decontamination and inactivation

Chemical inactivation efficacy data specific to LASV where chemical agent, concentration, and contact time with a log reduction of virus was not found during the literature review. There were no references to typical autoclave cycles for waste containing LASV (time and temperature).

### Conclusions

The need to rapidly diagnose LF infections is essential, because it is normally performed - in Core (BSL-2) laboratories using Enhanced Control Measures to manage risks associated with aerosol generation and waste management. Enhanced Control Measures in the laboratory include the use of a BSC for manipulation of samples suspected of containing virus, adherence to best practices to reduce the inadvertent generation of aerosols, and decontaminating wastes before removal from the BSC.

A risk-based approach may allow some LASV activities, such as diagnostics using molecular-based methods, to be performed at lower BSLs following the virus inactivation step. LASV inactivation using various reagents should be validated in-house before moving samples to a lower BSL. The same applies to requirements for validating decontamination methods before disposing of waste containing the virus.

Nosocomial outbreaks underscore the importance of using standard barrier protection by HCW. The WHO recommends using gloves, long-sleeved gowns, face shields or masks and goggles in the healthcare setting and when handling LF patient samples.

Although the United States and several other countries require the reporting of LASV LAI, until a global reporting policy and program are implemented, LAIs will likely remain under-reported.
